# Bioink Formulations for Bone Tissue Regeneration

**DOI:** 10.3389/fbioe.2021.630488

**Published:** 2021-02-05

**Authors:** Na Li, Rui Guo, Zhenyu Jason Zhang

**Affiliations:** ^1^School of Chemical Engineering, University of Birmingham, Birmingham, United Kingdom; ^2^Key Laboratory of Biomaterials of Guangdong Higher Education Institutes, Guangdong Provincial Engineering and Technological Research Centre for Drug Carrier Development, Department of Biomedical Engineering, Jinan University, Guangzhou, China

**Keywords:** bioink, formulation, bioprinting, hydrogel reinforcement, stimuli response, bone regeneration

## Abstract

Unlike the conventional techniques used to construct a tissue scaffolding, three-dimensional (3D) bioprinting technology enables fabrication of a porous structure with complex and diverse geometries, which facilitate evenly distributed cells and orderly release of signal factors. To date, a range of cell-laden materials, such as natural or synthetic polymers, have been deployed by the 3D bioprinting technique to construct the scaffolding systems and regenerate substitutes for the natural extracellular matrix (ECM). Four-dimensional (4D) bioprinting technology has attracted much attention lately because it aims to accommodate the dynamic structural and functional transformations of scaffolds. However, there remain challenges to meet the technical requirements in terms of suitable processability of the bioink formulations, desired mechanical properties of the hydrogel implants, and cell-guided functionality of the biomaterials. Recent bioprinting techniques are reviewed in this article, discussing strategies for hydrogel-based bioinks to mimic native bone tissue-like extracellular matrix environment, including properties of bioink formulations required for bioprinting, structure requirements, and preparation of tough hydrogel scaffolds. Stimulus mechanisms that are commonly used to trigger the dynamic structural and functional transformations of the scaffold are analyzed. At the end, we highlighted the current challenges and possible future avenues of smart hydrogel-based bioink/scaffolds for bone tissue regeneration.

## Introduction

There are a number of challenges associated with the current restoration methods for bone defects, such as autografts and allografts (Chiarello et al., 2013; Van De Vijfeijken et al., 2018), which include a limited supply of donor tissues, risk of complications, transplant rejection, and biocontamination (Vidal et al., 2020). Bone tissue engineering, an emerging route, has demonstrated a great potential over the past decades: replacing bone tissue with a three-dimensional (3D) living scaffold implant containing cells; using bone tissue extracellular matrix (ECM) mimics and the bioactive factors to activate the growth of cells (Qu et al., 2019). In here, the ECM mimics are commonly cytocompatible polymeric matrices, such as hydrogels, polyesters, and polymer-ceramic composites (Jakus et al., 2016; Rosales and Anseth, 2016; Nicolas et al., 2020), which require extensive research input from the material research community. It has been demonstrated extensively that hydrogels are greatly suitable for the biomedical applications due to their excellent biocompatibility and biodegradability, outstanding hydrophilicity, controllable permeability, and simple manufacturing methods (Fuchs et al., 2019).

A plethora of fabrication routes for bionic 3D scaffolds have been developed (Raeisdasteh Hokmabad et al., 2017; Chocholata et al., 2019; Haider et al., 2020), which include phase separation (Bailey et al., 2012), electrospinning (Thorvaldsson et al., 2013), freeze-drying (Wu et al., 2010), and salt leaching (Chiu et al., 2009). These strategies showed an excellent capability in producing a porous matrix with controlled surface morphology and adjusted processing variables, but sometimes a less controlled structure (Zhang et al., 2013). As a contrast, 3D bioprinting technique provides a fabrication process of scaffolds with complex and diverse geometry to evenly distribute cells and orderly release signal factors when cell-laden materials, known as bioink, are deployed (Bracaglia et al., 2017; Datta et al., 2017).

To regenerate a bone tissue, constructing a complex 3D structure with customized and enriched composition of ECM is one of the most indispensable initial steps (Dutta and Dutta, 2010). It is equally vital that the dynamic changes of the scaffolding can stimulate distinctive functions of tissue (Zhang et al., 2018), which requires some intrinsic mechanism(s) that can respond to external stimuli. To meet such technical requirements for tissue regeneration (Yang G. H. et al., 2019), 3D bioprinting technology evolved into four-dimensional (4D) bioprinting, whereby time is integrated as a processing parameter. Specifically, as the fourth dimension, time, changes (Kim S. H. et al., 2020), the geometric shape and behavior of a bionic scaffold are transformed, depending on the components' response to the surrounding environmental factors (Yang G. H. et al., 2019) and/or the internal function of the scaffold that progressively matures through the interaction with the cells (Wan et al., 2019). The technological advance offers not only the ability for biomimetic scaffold to respond immediately to any internal cell force stimulation and external factors, but also a gradual maturation and functional expression of the printed scaffold implants over time (Li et al., 2016; Wan et al., 2019). As such, stimuli-responsive 4D printed scaffolds, based on hydrogel, provide an unprecedented potential for bone tissue engineering (Murphy et al., 2013).

With a focus on the strategies concerning 3D reconstruction of bone tissue, in particularly the hydrogel-based bioink for hard tissue repair, this review starts with the technical requirements for bioprinting in terms of processability, resulting scaffold structure, and the bone repair function. A number of 3D printing processes are reviewed subsequently, comparing the advantages and limitations of each technologies, followed by the stimulus-responsive mechanism commonly used in 4D printing. Finally, some of the current challenges and potential improvements of hydrogel-based bioinks for future consideration are discussed.

## Property Requirements of Bioinks/Scaffolds

To 3D print a hard or elastic 3D hydrogel scaffold with orderly distributed components and a controlled geometry, bioink formulation with pre-defined rheological properties undergoes a complex manufacturing process. The following characteristics are therefore desirable for the bioinks:

1. Printability: It requires an appropriate viscosity range of the formulation and fast transition kinetics from sol to gel state (Kim M. K. et al., 2020). For nozzle-based extrusion bioprinting, bioinks are expected to be shear-thinning liquids that can be extruded in a laminar flow, whilst maintaining sufficient mechanical strength to self-support over multiple layers, with controlled geometry and porosity (Jakus et al., 2016). For example, by simply processing the sodium alginate/poly(acrylamide-co-acrylic acid) bio-ink formulation in a solution of pH 14, the viscosity and gel rate of the hydrogel can be adjusted to an improved 3D printability (Li et al., 2018).
2. Biocompatibility: Once it is ready, the scaffold would incorporate with the hosting biological environment, without triggering any adverse reactions, to actively promote the adhesion and proliferation of osteocytes. This would facilitate the formation of an extracellular matrix on the surface and in the pores of the scaffold, which gradually induce the development of a new bone tissue (Dong et al., 2009). In a recent report, the dental implants of minipig were grit-blasted with some ceramic particles, followed by acidic etching, to fabricate nano-structured surface. A very limited number of inflammatory cells were recorded after 4 and 12 weeks of implantation, which proves the biocompatibility of the implant (Hoornaert et al., 2020). Furthermore, the microenvironment within the scaffold is expected to encourage the formation of blood vessels in or around the implant within a few weeks after implantation, thereby providing a favorable condition for the transportation of nutrients, oxygen, and waste (Williams, 2008).
3. Mechanical properties: An appropriate network enhancement mechanism should form between the components of the scaffold to prevent material failure and minimize fracture under large deformation (Zhang et al., 2017). The mechanical properties of the fabricated scaffold should match those of the host bone, enabling a suitable load transfer. For example, Ratheesh and co-workers tried to add 15% w/v of inorganic bone particles, whose sizes are 150–500 and 0–500 μm, to 10 and 12.5% w/v methacrylated gelatin based bioink to enhance the biomechanical properties of the scaffold (Ratheesh et al., 2020).
4. Biodegradability: The printed scaffold is expected to degrade *in vivo* with time. Ideally, the degradation rate of scaffolds matches the production rate of ECM, whilst the degradation products have no side effects on the host (Sabir et al., 2009). For example, the mass loss of a waterborne polyurethane scaffold reached 35.62% after 90 days of degradation, while the fluorescence image showed that after 7 days of incubation, rabbit chondrocytes covered all waterborne polyurethane scaffolds (Feng et al., 2020).
5. Surface characteristics: The interface between the scaffold and the tissue to be repaired is critical to the success of a scaffold transplantation, with numerous reactions and interactions taking place. Therefore, the surface characteristics of a scaffold, such as porosity, wettability, and morphology, require significant attention. For instance, the diameter of the initially interconnected pores should maintain at least 100 μm, allowing nutrients and oxygen that are essential for cell survival to diffuse and the waste produced by the cells to transfer (Rouwkema et al., 2008). A pore size range of 200–350 μm was recommended for the growth of bone tissue (Murphy et al., 2010). Surface of the scaffold should be engineered to control cells spreading, proliferation, and differentiation. The morphology of the scaffold is also of great significance for anchoring cells and proteins on the surface (Haider et al., 2020).
6. Stimulus responsiveness: When the hydrogel is subjected to an external/internal stimuli, it can respond in a timely manner by changing its physiochemical properties. A reversible process is preferred (Lui et al., 2019).

**Figure 1:**
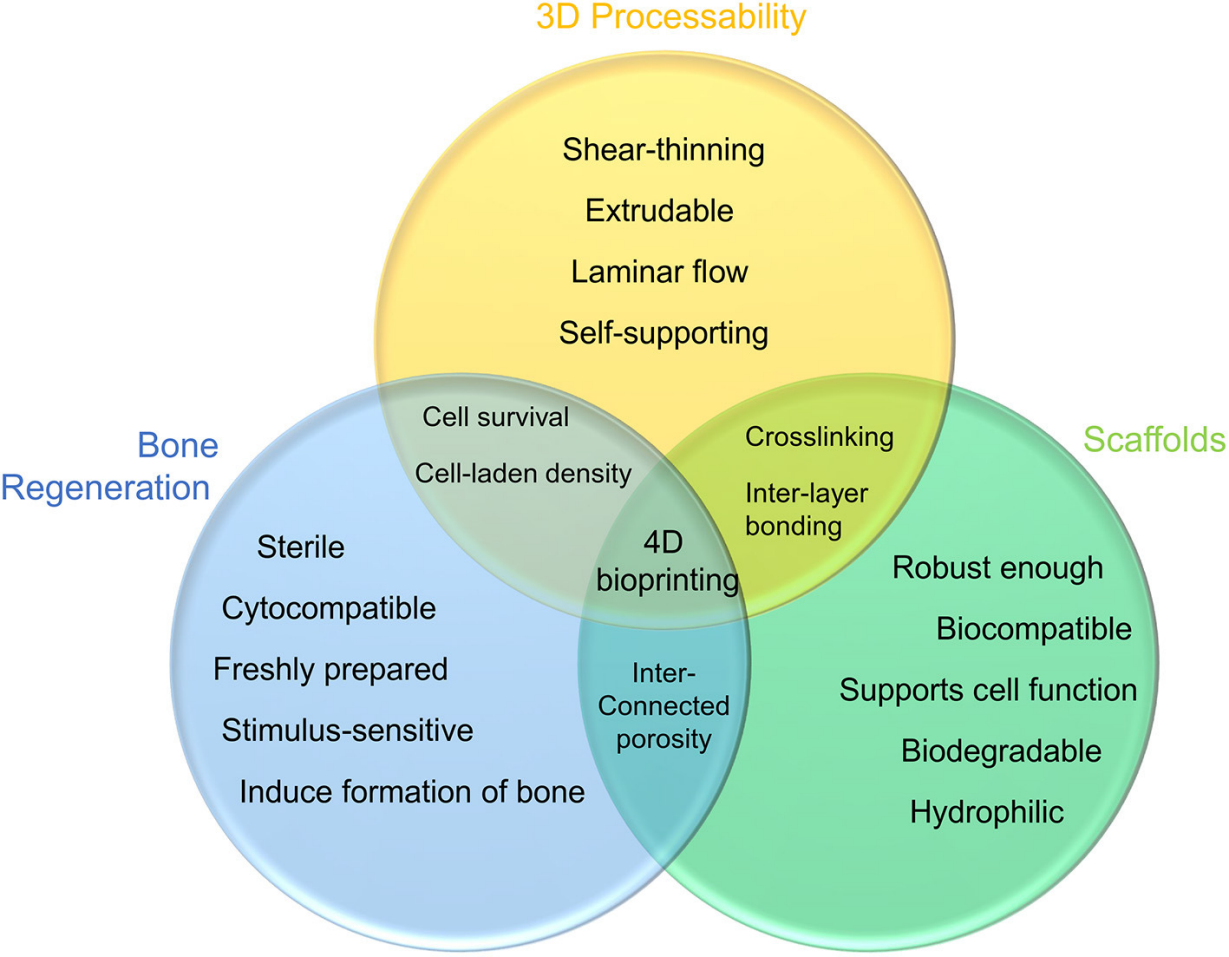
Requirements for bioink: the shear-thinning formulation is extruded in the form of laminar flow and forms desired geometry via its self-supporting ability (yellow); bioink can be crosslinked to enhance the mechanical strength of the hydrogel scaffold, whilst the biocompatibility and biodegradability promote cell diffusion and proliferation (green); to accomplish bone tissue repair, the scaffolding will induce new tissue growth and respond to external stimuli as the new bone matures (blue).

The properties of bioink and its resulting scaffolding outlined above do not exist independently at any specific stage. From the bioink in its liquid state, to the scaffold constructed, and finally to the functional implant, these characteristics are interrelated and could influence each other throughout the entire process that involves both physical and chemical transformations (Figure 1).

## Bioprinting Process

**Figure 2:**
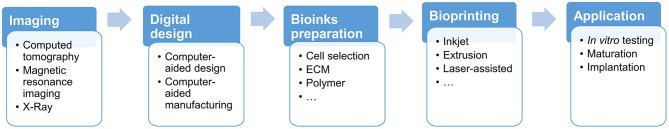
A typical process of bioprinting 3D scaffolding: imaging and digital design; preparation of bioinks formulation and cells; bioprinting a scaffold with defined geometry and structure; *in vitro* testing and transplantation.

As an advanced approach for bone tissue engineering, 3D bioprinting was developed upon the significant advances achieved in 3D printing technology, cell biology, and materials science/engineering in recent years. Figure 2 illustrates the overall bioprinting process, whereby bioactive scaffolds are fabricated via a layer-by-layer positioning of bioinks that commonly incorporate cells, bioactive factors, and biocompatible materials.

As the very first step, it is essential to establish the geometry and structure of the biomimic scaffold, upon examination of the impaired tissue. Computed tomography (CT) and magnetic resonance imaging (MRI) are the imaging technologies commonly used (Ozbolat and Gudapati, 2016) to establish the geometric requirements. Computer-aided design and manufacturing tools would then be deployed to construct the corresponding 3D structure. Composition of the bioinks will then be formulated, taking into account the desired characteristics specified in section Property Requirements of Bioinks/Scaffolds. Commonly used materials include biocompatible synthetic or natural polymers such as polycaprolactone and hyaluronic acid (Park et al., 2014), bioactive glass (Murphy et al., 2017), calcium phosphate (Ahlfeld et al., 2018), and natural decellularized fibers (Pati et al., 2014). It is worth noting that cells encapsulated in the bioink can be derived from autologous or allogeneic sources (Murphy and Atala, 2014).

**Figure 3:**
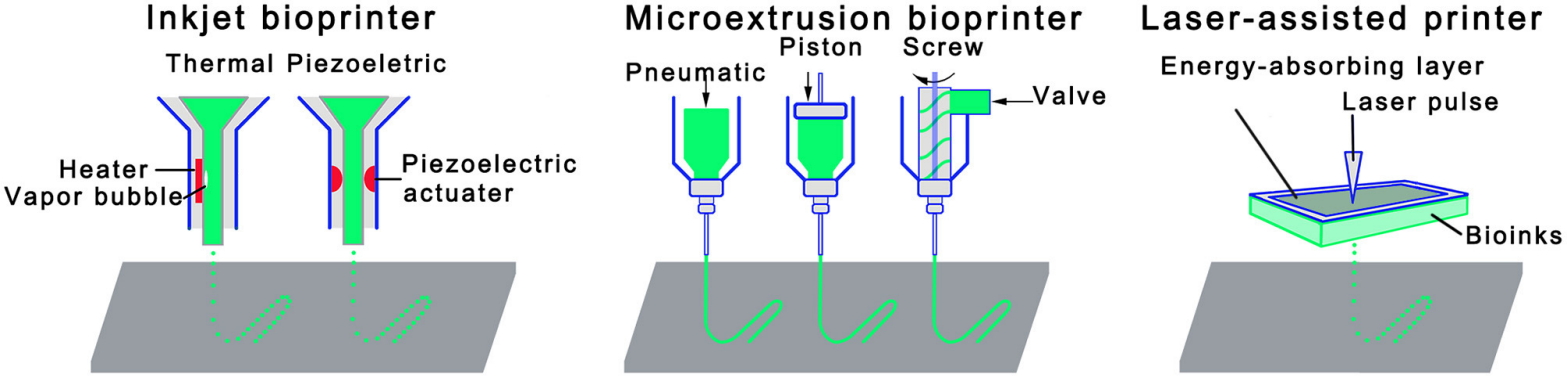
Schematic diagrams of three primary bioprinting technologies: inkjet bioprinting, extrusion bioprinting, and laser-assisted bioprinting.

As shown in Figure 3, a variety of bioprinting technologies have been developed to deposit bioink on a supporting substrate with high spatial resolution. An inkjet bioprinter can convey small droplets of formulation over predefined locations by either thermal (Cui et al., 2010, 2012) or acoustic mechanisms (Xu et al., 2008; Fang et al., 2012). The heat acts as a driving force to evaporate the deposited ink into bubbles that burst and cause the ink to be ejected (Gomes et al., 2012). Piezo actuators eject ink from nozzles by offering a transient pressure (De Jong et al., 2006). Although thermal inkjet printing has several technical advantages such as high printing speed/efficiency and low operating cost, there are serious limitations, for example, the droplets cannot be completely uniformly transferred, resulting in an irregular droplet size or shape. In addition, cells and formulations that are not heat-resistant or mechanically resistant may be compromised. Piezoelectric inkjet printers have shown the capability to address the above limitations associated with the thermal one (Nakamura et al., 2005; Saunders et al., 2008), but there have been concerns with regard the potential damage to the cell membrane and lysis induced by acoustic waves at 15–25 kHz frequencies (Cui et al., 2012).

Amongst all 3D bioprinting technologies developed, extrusion bioprinting is arguably the most widely used one due to its exceptional capability of accommodating a broad spectrum of fluids whose viscosities range from 30 mPa s^−1^ to just under 6 × 10^7^ mPa s^−1^ (Jones, 2012). In this process, bioink formulation is dispensed to large hydrogel filaments on the substrate either pneumatically (Chang et al., 2011) or mechanically (piston- or screw-driven) (Jakab et al., 2006; Visser et al., 2013). Since the bioink is subjected to a strong shear stress when flowing through the nozzle, its rheological characteristics, in particularly the shear thinning characteristics, is critical to the processing condition (Shin et al., 2019). Compared with inkjet bioprinting, an obvious technical advantage offered by the extrusion bioprinting is a high cell deposition density, as summarized in Table 1. However, survival rate of cells is not as satisfying as that with inkjet bioprinting because cells may be subjected to an increased shear stress and mechanical pressure during the dispensing process. Some countermeasures, such as reducing the extruding pressure, increasing the nozzle size, are necessary to improve cell survival rate, which would compromise the printing speed and resolution.

**Table 1:** Comparison of the advantages and disadvantages of inkjet bioprinting, extrusion bioprinting, and laser-assisted bioprinting.

**Bioprinting technologies**	**Advantages**	**Disadvantages**
Inkjet bioprinting	Low operating cost High efficiency	Non-uniformity of droplet size Inaccurate deposition location; Heat damage to cell behaviors Cell viability >85% Requirement for low viscosity bioinks
Extrusion bioprinting	Wide bioink printing viscosity High cell deposition densities	Low cell viability Low resolution Low printing speed
Laser-assisted bioprinting	High density of cell encapsulation Accurate and fast printing	High operating cost Complicated preparation process The trace metallic residues

The third fabrication strategy is laser-assisted bioprinting that is consisted of three primary components: a pulsed laser source; a ribbon coated with a metallic absorbing layer (e.g., gold or titanium) and a layer of bioink; and a receiving substrate such as quartz disk (Guillemot et al., 2011). During the construction of a scaffold, a pulsed laser beam scans over a ribbon, which triggers vaporization of the sacrificial absorbing layer. The resulting vapor bubbles would initially collapse on the surface of the absorbent layer, with no ejection. However, as the energy accumulates in the bubble, the pressure in the bubble increases before it bursts, expelling the bioink onto the receiving substrate (Guillemot et al., 2010). The technology could accommodate bioinks of various viscosities, and can achieve a high density of encapsulated cells and cell survival rate. There are also limitations such as the complex preparation of the ribbon, a trace amount of metallic residues in the scaffolds, and the high production cost (Murphy and Atala, 2014).

Following the 3D printing process, a bioink formulation will experience a sol-gel transformation to form a stable scaffold, no matter which printing technology is used. To ensure the survival rate of the cells, the prepared scaffold must be matured in a bioreactor for a period of time, and undergo a series of necessary *in vitro* tests before transplantation.

## Strategies for Hydrogel Bioink Reinforcement

**Figure 4:**
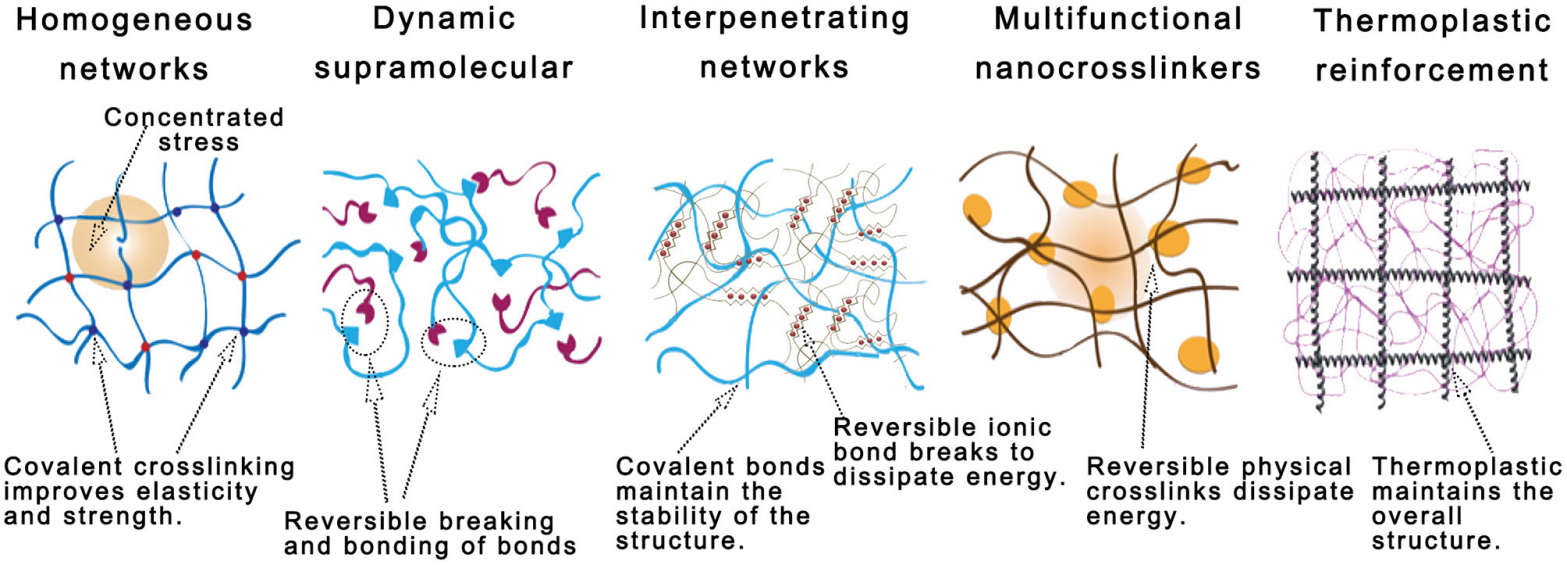
Schematic diagrams of five established mechanical reinforcement techniques for hydrogel-based bioinks: polymer functionalization; supramolecular-reinforced hydrogels; interpenetrating networks (IPNs); nanocrosslinkers; and thermoplastic reinforcement.

Once being extruded from the nozzle, bioink is expected to crosslink rapidly and solidify to maintain its geometrical integrity with an enhanced mechanical property, of which the kinetics was considered the most important processing parameter during the early stage of 3D bioprinting development. Biocompatibility, another critical factor, considers the biological activity and toxicity of each component in the bioink formulation. With the advancement of bioprinting technology, reinforcement strategies of bioink formulations received a great attention, of which the principle and mechanisms will be reviewed in this section (Figure 4).

### Polymer Functionalization and Homogeneous Hydrogel Networks

Of the many natural polymers used in bioink formulations, some can form a physically crosslinked network through reversible conformational transitions. However, the formed network has a low bonding energy: changes in the surrounding environment can easily disrupt the conformation of the polymer chains, resulting in unsatisfactory mechanical properties (Rastogi and Kandasubramanian, 2019). To enhance the crosslinking ability of bioink formulations, a common strategy is to incorporate some new functional moieties or modify the end/side groups of the natural polymers, which could introduce additional or specific crosslinking sites and consequently promote the formation of a homogeneous network. With a uniformly distributed load, such network can minimize a localized stress concentration and, thereby improve the fracture toughness of the material (Sakai et al., 2008).

One of the common approaches used is to modify the side groups of polymers with methacrylate moieties, which has been applied to many natural polymers, including gelatin (Boere et al., 2014; Yin et al., 2018), alginate (Gao and Jin, 2019), hyaluronic acid (Skardal et al., 2010b), collagen (Chen et al., 2019), and kappa carrageenan (Mihaila et al., 2013). The modified polymers are equipped with carbon-carbon double bonds in the side chain, which can be easily crosslinked by a light source of different wavelength in the presence of photo-initiators. In a recent work, methylcellulose/gelatin-methacryloyl (MC/GelMA) bioink demonstrated an outstanding shape integrity (Rastin et al., 2020): MC provided the bioink formulation an excellent processability whilst GelMA was used not only to form a covalent crosslinked network through light radiation, but also to establish the physical interaction with the MC, improving the mechanical strength of the formed hydrogel. The addition of GelMA to pure MC can significantly improve the critical shear stress required to initiate the viscous flow of the hydrogel, with a progressive rise in yield stress from 1356 ± 43 Pa (for pure MC) to 2354 ± 53 Pa (MC/GelMA hydrogel).

Click reactions, particularly thiol-ene click reactions (Lowe, 2010), demonstrated exceptional advantages, e.g., ease of use, high efficiency, reliability, and high selectivity (Cengiz et al., 2017), in synthesizing functional polymers or preparing polymers with topological structure for surface modification and biopharmaceutical applications (Yigit et al., 2011). For example, norbornene-modified hyaluronic acid underwent a photo-crosslinking reaction with the tetrathiol (Wang et al., 2018), and subsequently, the rheological measurements showed that the covalent networks improved the hydrogel mechanics whereby its storage modulus increased from 500 Pa to 5 kPa. Moreover, the Young's modulus of the hydrogel was enhanced from 1 to 3 kPa after photocuring.

It is feasible to develop a homogeneous network by combining two types of symmetrical multi-arm polyethylene glycols (PEG) whose end groups can react with each other. There are a number of flexibilities offered by PEG: easily changed molecular configuration, number of branches, type of end groups, and length of the arms, which offer a great platform for hydrogel reinforcement (Rutz et al., 2015) and for constructing a uniform network. Sakai and colleagues (Sakai et al., 2008) reported that the compressive strength of a gel network, once reacting two four-arm PEGs of the same size with amino and ester groups respectively, was significantly greater than that of agarose gel or acrylamide gel under the same network concentration. At a concentration of 120 mg/mL, the maximum breaking stress (9.6 MPa) of such PEG gel matched that of natural articular cartilage (~6–10 MPa). To summarize, strategies have been developed and demonstrated to significantly improve the mechanical properties of bioprinted scaffold, in terms of ductility, malleability, and fracture toughness, by fabricating a hydrogel network that can distribute the applied stress evenly.

### Dynamic Homogeneous Supramolecular Network

Enabled dynamic chemical bonds and/or physical interactions (Morgan et al., 2020), polymer can self-assemble and form a dynamic homogeneous supramolecular hydrogel.

The prominent dynamic covalent chemical reactions currently used include reversible diels-alder reactions, disulfide exchange, boronate ester formation, and aldimine formation (Morgan et al., 2020). The nature of such dynamic bonds is attributed to the combination of thermodynamically stable but kinetically unstable interactions (Jin et al., 2013). Reversible physical interactions include hydrogen bonding, ionic bonding, π-π stacking, hydrophobic interactions (Webber et al., 2016), coordination bonds (Zheng et al., 2016), and non-covalent guest–host interactions (Loebel et al., 2017). During the self-assembly process of the monomers that involve the dynamic interactions aforementioned, the inherent dynamicity does not facilitate the formation of a mechanically strong polymer structure (Mann et al., 2018), but enable the long chain polymer to entangle with each other if they are uniformly bound to a macromolecular backbone, forming macromolecular monomers initially. Such molecular configuration could reduce the free volume of chains, confine the migration ability of polymer chains, and improve the stability of the supramolecular network (Morgan et al., 2020). When being deformed, these dynamic interactions are constantly broken but reformed to mitigate a permanent disruption to the molecular network.

**Figure 5:**
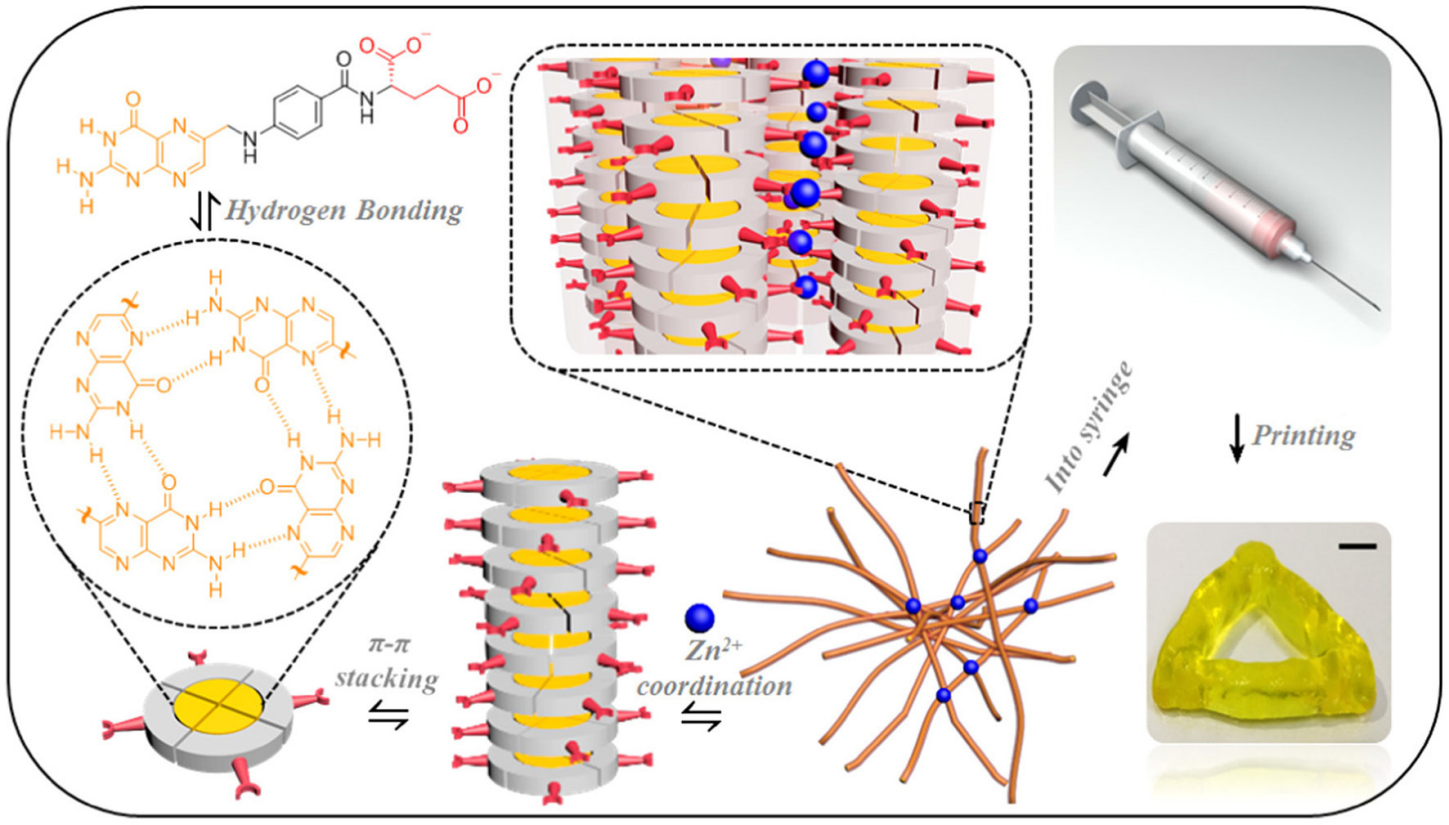
A folic acid fibril network formed through a series of physical interactions. Reprinted with permission from Liu et al. (2018). Copyright (2018) American Chemical Society.

Figure 5 presents a number of intermolecular interactions, e.g., hydrogen bonding of petrin rings in the molecular structure, π-π stacking, and zinc ions coordination, that were used to develop an injectable fibril network based on folic acid, a natural small molecule (Liu et al., 2018). The bioink formulation exhibited a shear-thinning characteristic during the printing process, and was able to self-heal immediately after printing due to the dynamic nature of the interactions. When the molar ratio of folic acid to zinc ions and the total concentration of the coordination system were increased, it was found that the mechanical properties of the hydrogel were improved significantly whilst maintaining the excellent printability. For example, the elastic modulus of such fibril network was improved by ~5 orders (from 10 to 10^6^ Pa) when changing the molar ratio of folic acid to zinc ions from 1.7 to 2.0. The adjustable mechanical properties could be invaluable to suit the requirements for repairing various biological tissues from cartilage to cancellous bone. Moreover, the simple preparation, excellent printability, biocompatibility, and mechanical properties of supramolecular hydrogels provide an excellent platform in applications such as drug delivery.

**Figure 6:**
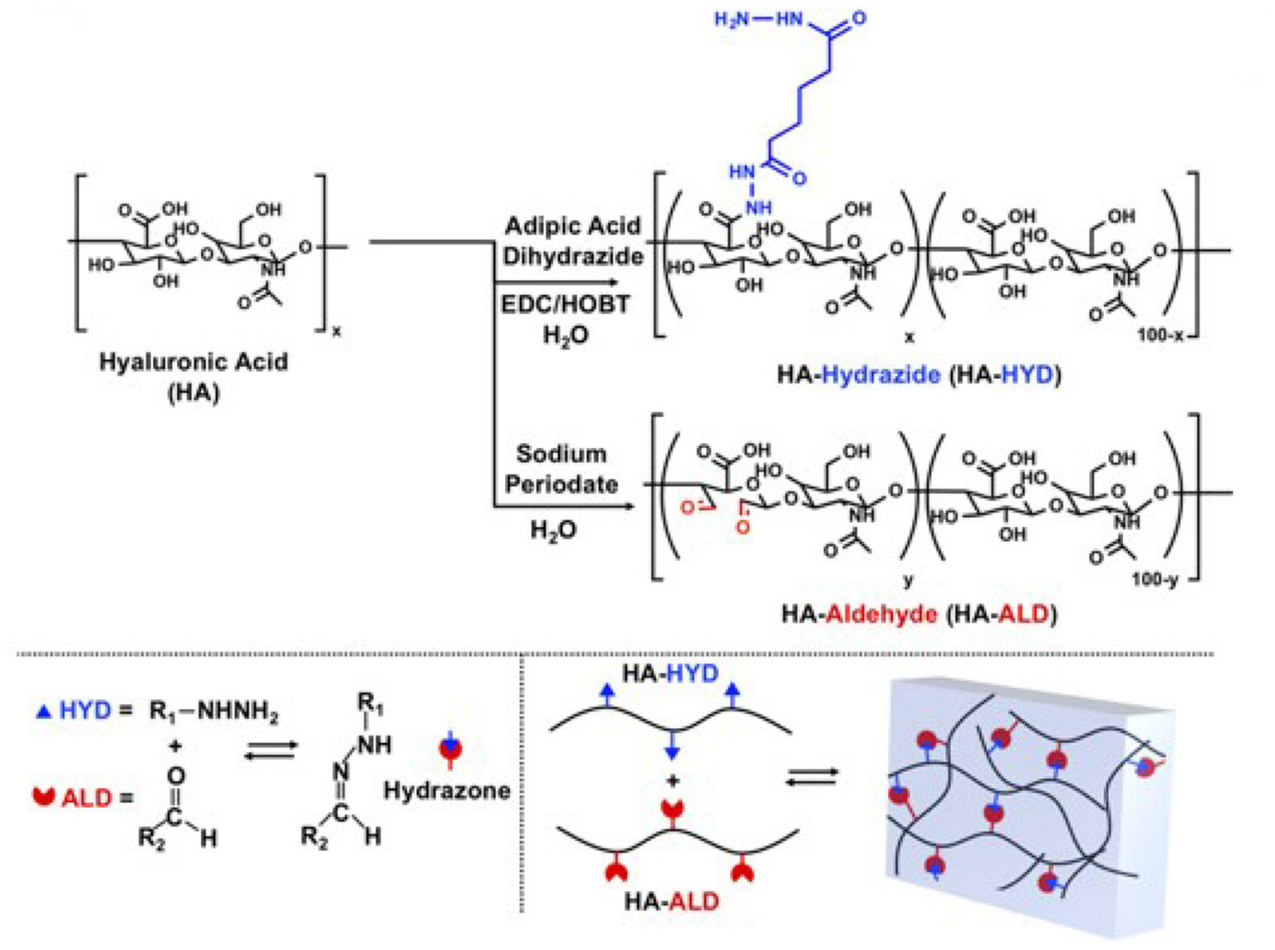
Schematic diagram of the hydrazide and aldehyde groups modified hyaluronic acid macromonomer and dynamic covalent network. Reprinted with permission from Wang et al. (2018). Copyright (2018) John Wiley and Sons.

Wang and colleagues (Wang et al., 2018) developed a bioink formulation that was enriched by dynamic covalent hydrazone bonds: hyaluronic acid was modified by hydrazide and aldehyde groups through amidation reaction and oxidation reaction, respectively (Figure 6). Once being exposed to an aqueous environment, the hydrazine groups can react reversibly with aldehydes to form dynamic covalent bonds. Rheological tests showed that the hydrogel exhibited the preferred shear thinning, self-healing, and injectable properties. Whilst maintaining an equal ratio between the two types of hyaluronic acid, an increased total mass fraction of the polymer (from 1.5 to 5 wt%) improved the shear modulus from c.a. 500 to 6,000 Pa, and Young's modulus to 15 kPa. However, the increased concentration of hyaluronic acid limited the self-healing efficiency of the hydrogel. It is worth noting that the viability of fibroblasts encapsulated in the hydrogel remained at a high level for each mass fraction. To further improve the mechanical properties of the hydrogel, click chemistry of norbornene-modified HA was used to generate a second network. The resulting hybrid hydrogel network was shown with an increased elastic modulus, a significantly reduced degradation rate, and a prolonged lifespan. This study demonstrates that different enhancement strategies could be deployed together to address the limitations associated with one particular crosslinking reaction, which expands the available options in developing a bioink formulation.

### Interpenetrating Networks (IPNs)

IPNs consist of two polymer networks that can entangle through covalent bonds or random physical interactions. Based on the nature of the interactions, IPNs can be divided into double networks (DNs), where each network is connected by covalent bonds, and ion-covalent entanglement networks (ICEs), where two networks rely on ionic bonds and covalent bonds, respectively (Chimene et al., 2020a).

**Figure 7:**
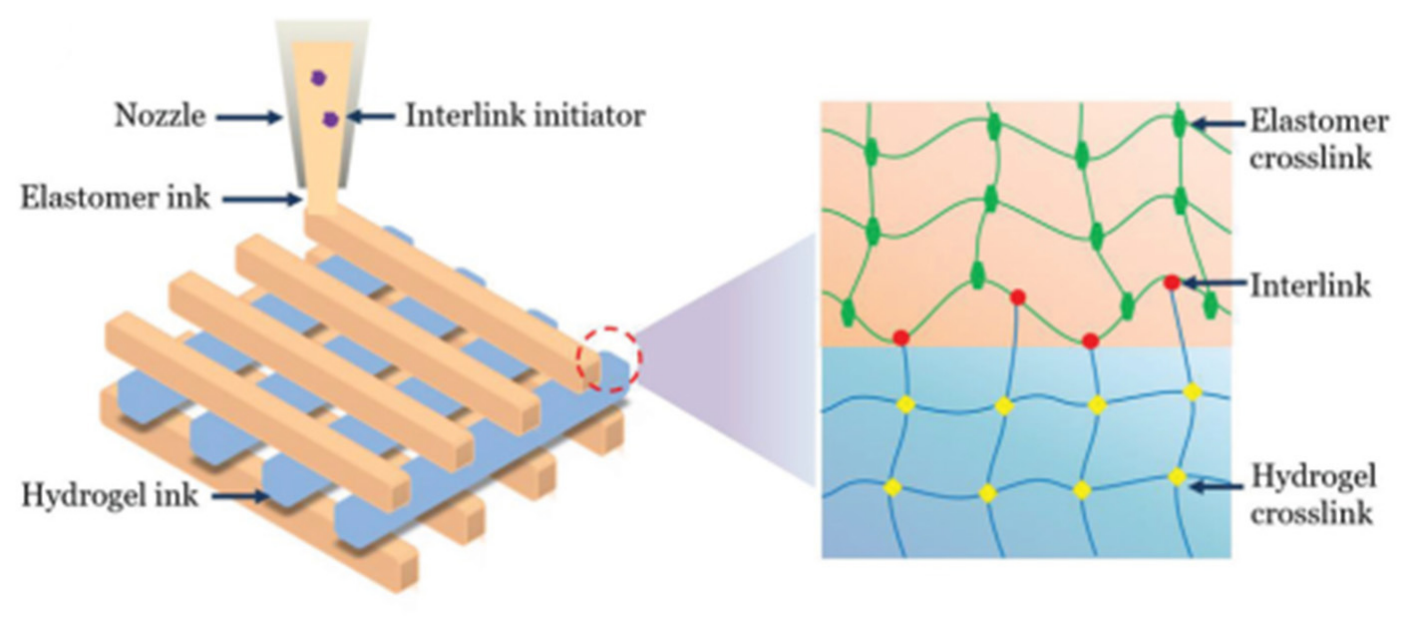
Schematic diagrams of integrated double networks through interlink initiator. Reprinted with permission from Yang H. et al. (2019). Copyright (2019) John Wiley and Sons.

The extensive presence of covalent bonds ensures that there is a substantial bond energy within the DNs. However, a long bonding time is required for the DNs, which might compromise their self-support ability following a bioprinting process. The interpenetrating networks of long-chain and short-chain molecules equip the DNs an excellent elasticity under deformation of up to 50%. The short chain network in the DNs would collapse first to dissipate energy, followed by the disintegration of the long chain network when the DNs undergoes a large deformation. Since the rupture of the DNs network is irreversible, their overall mechanical properties are primarily determined by the long-chain network that has a better elasticity than the short-chain network provides. For example, two networks, a hydrogel network and a hydrophobic elastomer network containing interlink initiators, were printed simultaneously (Yang H. et al., 2019): the initiators can induce covalent bonds between the two separated networks whilst each network is crosslinked during the curing process, which integrates the two networks together (Figure 7). It is worth noting that the interlink initiators were not deposited on the surface of the elastomer network, but evenly distributed within the elastomer network. The integrated structures provide an exceptional fracture energy (~10,000 J m^−2^ for the hydrogel and c.a. 6,000 J m^−2^ for the elastomer) as well as a high adhesion energy, above 5,000 J m^−2^. The prepared double network structure can also withstand swelling.

In comparison with the dual networks aforementioned, ICEs systems have a greater potential in improving the mechanical properties of 3D printed hydrogel scaffolds because they are composed of two network structures with dissimilar properties (Zhao, 2014). In here,

- The ionic cross-linked network is tightly arranged, with bonds formed via physical attraction of positive and negative charges, which has a fast bonding kinetics. However, the weak bonds formed are sensitive to changes in the external environment, such as temperature and ion concentration, which underpins the instable and reversible nature of such network.
- The covalent cross-linked network is a flexible network with loose mesh and excellent ductility. It shows an excellent stability due to its high bonding energy, but cannot be recovered once broken (Gong, 2010). The advantage of ICEs over DNs is that the breakup of the sacrificial bonds is temporary. Upon external deformation, the tightly cross-linked ionic bonds break due to their brittleness, resulting in a series of microscopic segments, but the soft covalent network could use the viscous dissipation mechanism to prevent such microcracks from developing into macrocracks (Fuchs et al., 2019). If the external stress disappears at this point, ionic bonds could reform to repair the cracks. However, the cracks would likely cascade across the material if the stress gradually increases to the point where the covalent cross-linked network fails.

**Figure 8:**
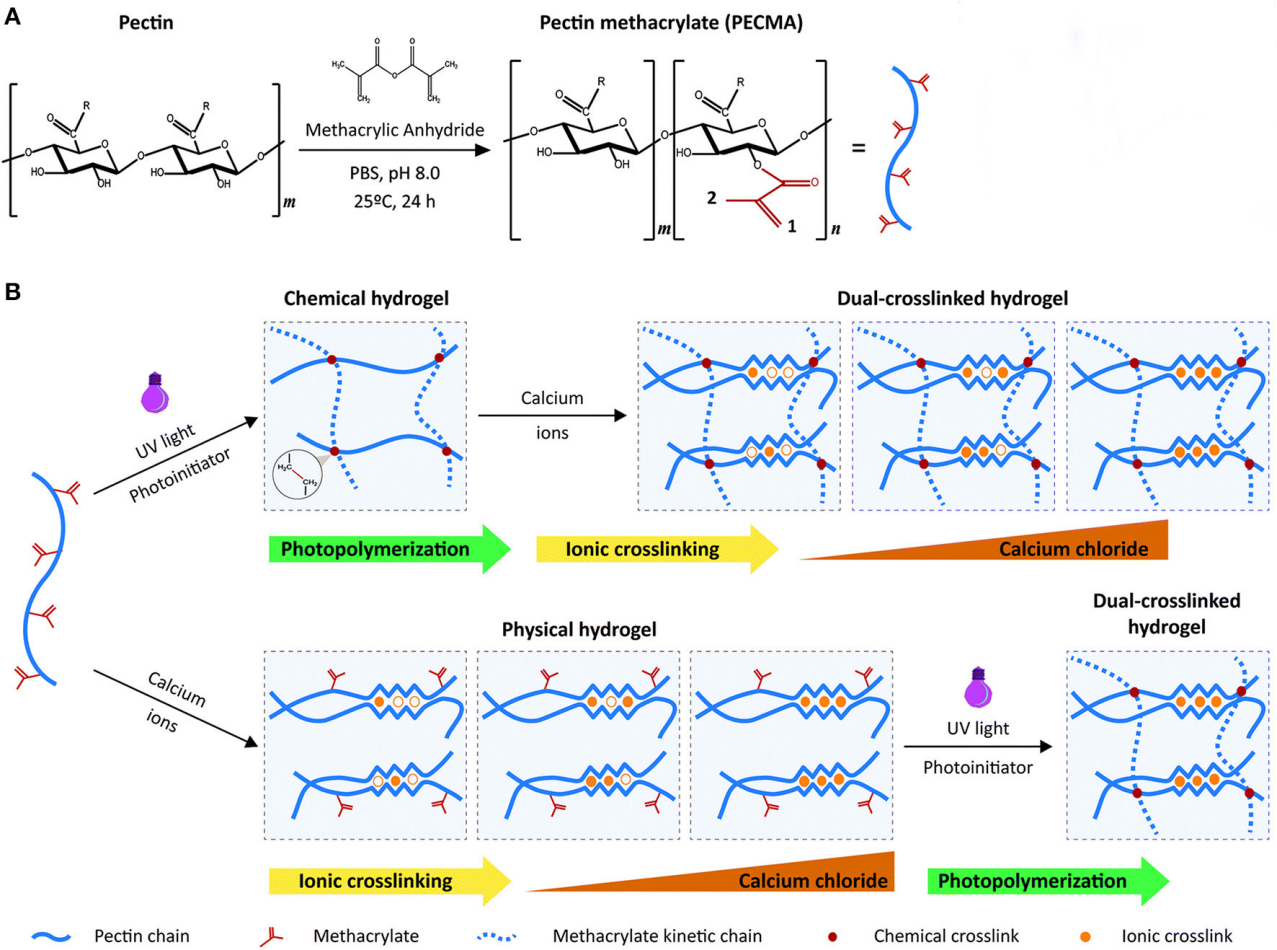
**(A)** Modification method of single-component pectin bioink; **(B)** Strategy of preparing ICEs hydrogels. The order of implementation of ionic crosslinking and photopolymerization does not affect the formation of the ICEs hydrogels. Reprinted with permission from Pereira et al. (2018). Copyright (2018) Royal Society of Chemistry.

In a recent report, Pereira et al. (2018) used pectin, an anionic heteropolysaccharide, as a sole bioink material to prepare an ICEs hydrogel. With the ability of calcium-mediated ionic gelation, pectin was modified via methacrylation, and subsequently crosslinked chemically by UV radiation to prepare a covalent network. As illustrated in Figure 8, the modified pectin retained the ability to form a physically crosslinked hydrogel, allowing the design of ICEs hydrogel in which the chemical cross-linking and ionic crosslinking of the modified pectin can be performed in any order. Several processing parameters, such as the degree of methacrylation, UV exposure time, and polymer concentrations, could affect the mechanical properties of the final ICEs hydrogel. Lastly, a base-catalyzed thiol-Michael addition click reaction was carried out to attach cell adhesion moiety (RGD sequence) to the pectin backbone, which would improve the biological inertness of the pectin network. Results showed that the pectin-based hydrogel had cellular response characteristics, facilitating the printed skin fibroblasts to secrete new ECM. The 3D structured hydrogel, prepared with a single-component bioink formulation, has many potential applications in skin tissue engineering. It was also pointed out that the initial concentration of the bioink and the light irradiation time need to be increased if the formulation were to be used for any future bone tissue regeneration applications.

### Multifunctional Nanocrosslinker

Incorporating nanocrosslinkers in the bioink formulations is a prominent method in enhancing hydrogel scaffold: with a large specific surface area, a single nanocrosslinker is able to interact with multiple polymer chains simultaneously (Fathi-Achachelouei et al., 2019). The amplified adhesion is primarily governed by physical interactions, e.g., hydrogen bonds, ionic bonds, electrostatic interactions, but can be actuated by chemical covalent cross-linking where appropriate/needed (Chimene et al., 2020a).

Once an external load is applied to a hydrogel containing nanocrosslinkers, a small fraction of polymer chains adsorbed on the nanocrosslinkers is forced to desorb and dissipate energy. However, such separation at molecular scale does not seem to have a notable impact on the overall structure of the hydrogel network. In addition, the detached polymer chains will re-adsorb onto the nanocrosslinker via physical interaction according to the principle of proximity (Zhalmuratova and Chung, 2020). The dynamic nature of the molecular interactions between nanocrosslinkers and polymers not only enhances the overall mechanical strength, but also improves the fatigue resistance of the network.

Nanocrosslinkers can also help to improve the rheological properties and processibility of the hydrogel, introduce responsiveness to stimulus such as electric or magnetic field, and promote tissue regeneration processes, such as bone formation and mineralization (Hasan et al., 2018; Tang et al., 2018). The versatility of nanocrosslinkers is primarily determined by the characteristics of the nanomaterials, such as shape, chemical nature, size, structure, and surface charge. According to the difference in their shapes, nanocrosslinkers with excellent biocompatibility can be divided into the following categories:

- dot-shaped nanoparticles (NPs), such as hydroxyapatite NPs, Fe_3_O_4_ magnetic NPs, gold, and silver NPs (Skardal et al., 2010a)
- nanofibers (Lu et al., 2019) and nanotubes, such as carbon nanotubes (CNTs), and nanocellulose
- layered low-dimensional nanosheets, such as nanoclay, graphene, and graphene oxide.

**Figure 9:**
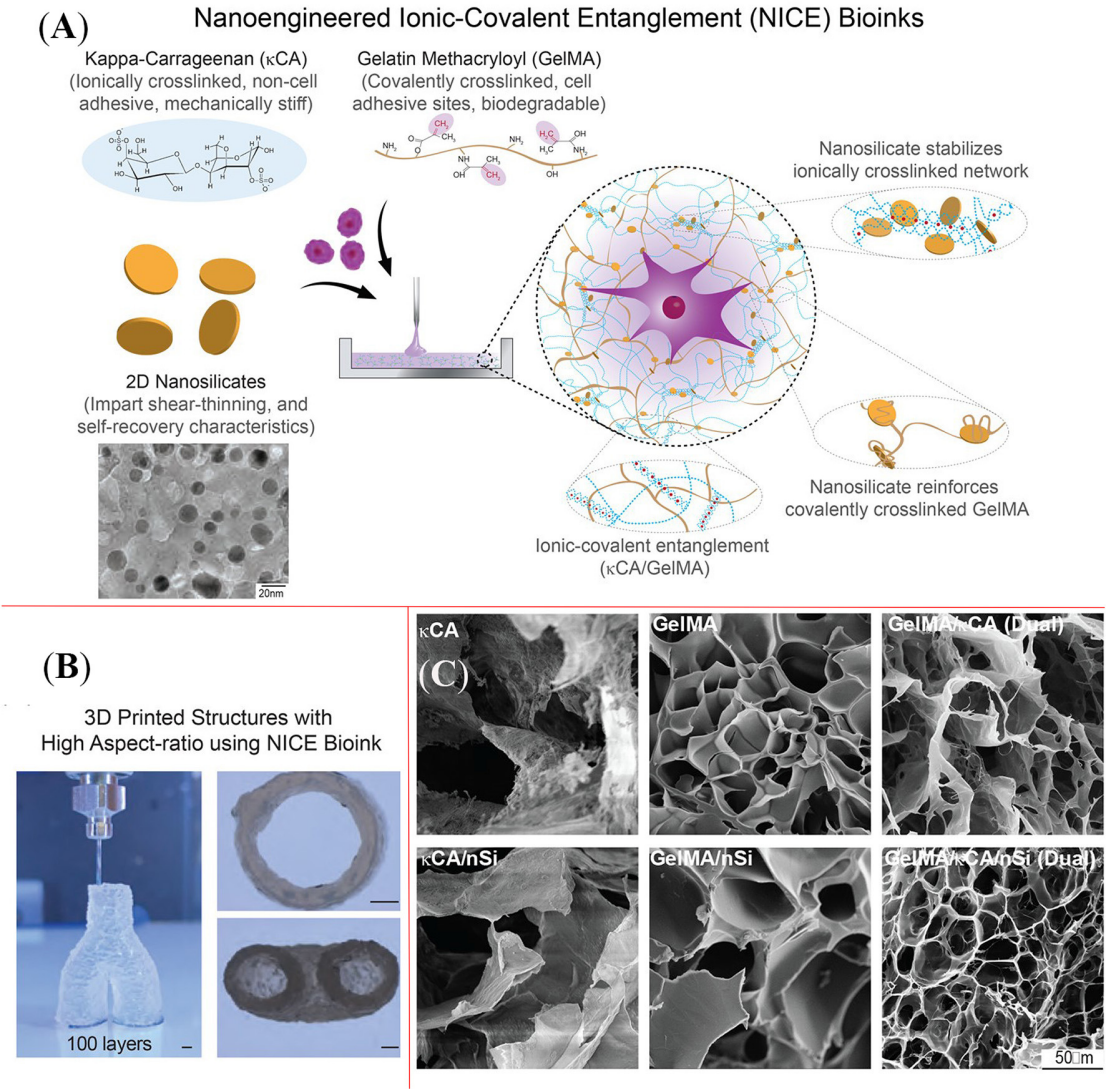
**(A)** Schematic diagram shows the function of nanosilicate, gelatin methacryloyl, and kappa-carrageenan in nanoengineered ioni-covalent entanglement (NICE) bioink system; **(B)** NICE bioink was capable to print a scaffold with hing aspect-ratio, and the height of the construct reached 3 cm; **(C)** Comparison of pore structure of various bioink formulations. Reprinted with permission from Chimene et al. (2018). Copyright (2018) American Chemical Society.

Example used here to demonstrate the capability of nanocrosslinkers for hydrogel reinforcement is a nanoclay, known as nanosilicate or Laponite (Chimene et al., 2018, 2020b), that is a disc-like 2D nanoplatelet with good solubility in water and satisfactory biocompatibility. Possessing negative charge on its surface but positive charge around the edge, nanosilicate could interact easily with polyelectrolyte. Figure 9A presents a nanosilicate (nSi) based ICEs bioink formulation (NICE) that contains gelatin methacryloyl (GelMA) and kappa-carrageenan (κCA) (Chimene et al., 2018). It appears that the addition of nanosilicates could increase the viscosities of formulations based on GelMA and κCA alone: NICE bioink formulation was used to prepare scaffolds up to 3 cm tall (150 layers), which is 10 greater than the maximum height constructed using the other single or binary bioink formulations (GelMA, GelMA/nSi, κCA, κCA/nSi, GelMA/κCA), as shown in Figure 9B. The substantially improved mechanical properties are likely attributed to a synergistic effect between nanoclay and ICEs, which is much greater than the effect by the individual factor. Unlike the conventional hydrogel enhancement approaches, e.g., increasing the crosslinking density and overall polymer concentration, such combined effect can construct a tough but elastic 3D scaffold without compromising the porosity of the hydrogel (Figure 9C). An additional benefit reported is that the nanosilicates were able to stimulate the intrachondral differentiation of human mesenchymal stem cells and the accumulation of extracellular matrix.

Over the past few years, nanocellulose that is extracted and/or separated from plants and bacteria has received much attention due to its sustainable resource, non-toxicity, high specific surface area and aspect ratio, and excellent mechanical properties. Based on its dimensional characteristics, nanocellulose can be divided into several groups: cellulose nanocrystals (CNC), bacterial nanocellulose (BNC), cellulose nanofibers (CNF), microfibrillated cellulose (MFC), cellulose microfibrils, and nanowiskers (Curvello et al., 2019). A novel bone tissue substitute was developed by adding hydroxyapatite NPs and MFC in turn to a collagen hydrogel (He et al., 2018): the hydroxyapatite NPs could facilitate the interfacial adhesion with the tissues in contact and promote the proliferation of osteoblasts, whilst the hydrogen bonds available abundantly on the MFC promote the formation of a network, which enhances the mechanical properties and degradation properties of the scaffold. This was evidenced by an exceptional compressive strength of the prepared nanocomposite scaffold (>28.25 MPa) that is greater than what a sponge bone could offer (1–20 MPa) (Gibson, 1985) when the MFC content was over 15%. The work is one of the many recent attempts in demonstrating that the low-cost and sustainable materials such as MFC have a great potential in the bioprinting applications.

Distribution of nanocrosslinkers within a formed hydrogel scaffold is a critical factor: homogeneously distributed nanoparticles could help to minimize the formation of localized microcracks upon externally applied stress (Chimene et al., 2015). However, hydrophobic nanocrosslinkers tend to form aggregates, instead of interacting with the macromolecules involved, which might compromise the overall stability and mechanical properties of the hydrogel. One of the possible solutions to address such issue is using amphiphilic compounds. A comb-like polymer was included in a bioink formulation containing both hydrophobic CNTs and hydrophilic bacterial cellulose network (BC) to increase the binding energy and improve the overall mechanical properties of the hydrogel (CNT-BC-Syn) (Park et al., 2015). This approach not only improved the dispersibility of the CNTs in the hydrogel, enhanced the structural integrity and mechanical properties of the hydrogel, but also exhibited excellent osteoinductivity and osteoconductivity.

### Thermoplastic Reinforcement

Naturally, thermoplastics possess several inherent advantages as a candidate for tissue scaffolding, such as superior mechanical strength and excellent processibility for complex-shaped objects. However, they are not suitable for bioink formulation due to their hardness and high melting temperature that is not suitable for the cell-containing hydrogel (Kim et al., 2016).

To address these technical challenges, a novel strategy was developed whereby the thermoplastic was processed in a separate chamber and printed with hydrogel bioinks in either layers or pre-defined patterns, which results in a two-phase structure where the thermoplastic component acts as the backbone. To minimize any potential damage to the cells by the thermoplastics, only the ones with low melting temperatures were considered. Of a broad range of biocompatible and biodegradable synthetic polymers, polyglycolic acid (PGA) (Day et al., 2004), polylactic acid-glycolic acid copolymer (PLGA) (Moran et al., 2003), polylactic acid (PLA) (Wang et al., 2016), polycaprolactone (PCL) with a melting temperature of about 65°C (Kang et al., 2016) have been explored in the previous studies.

**Figure 10:**
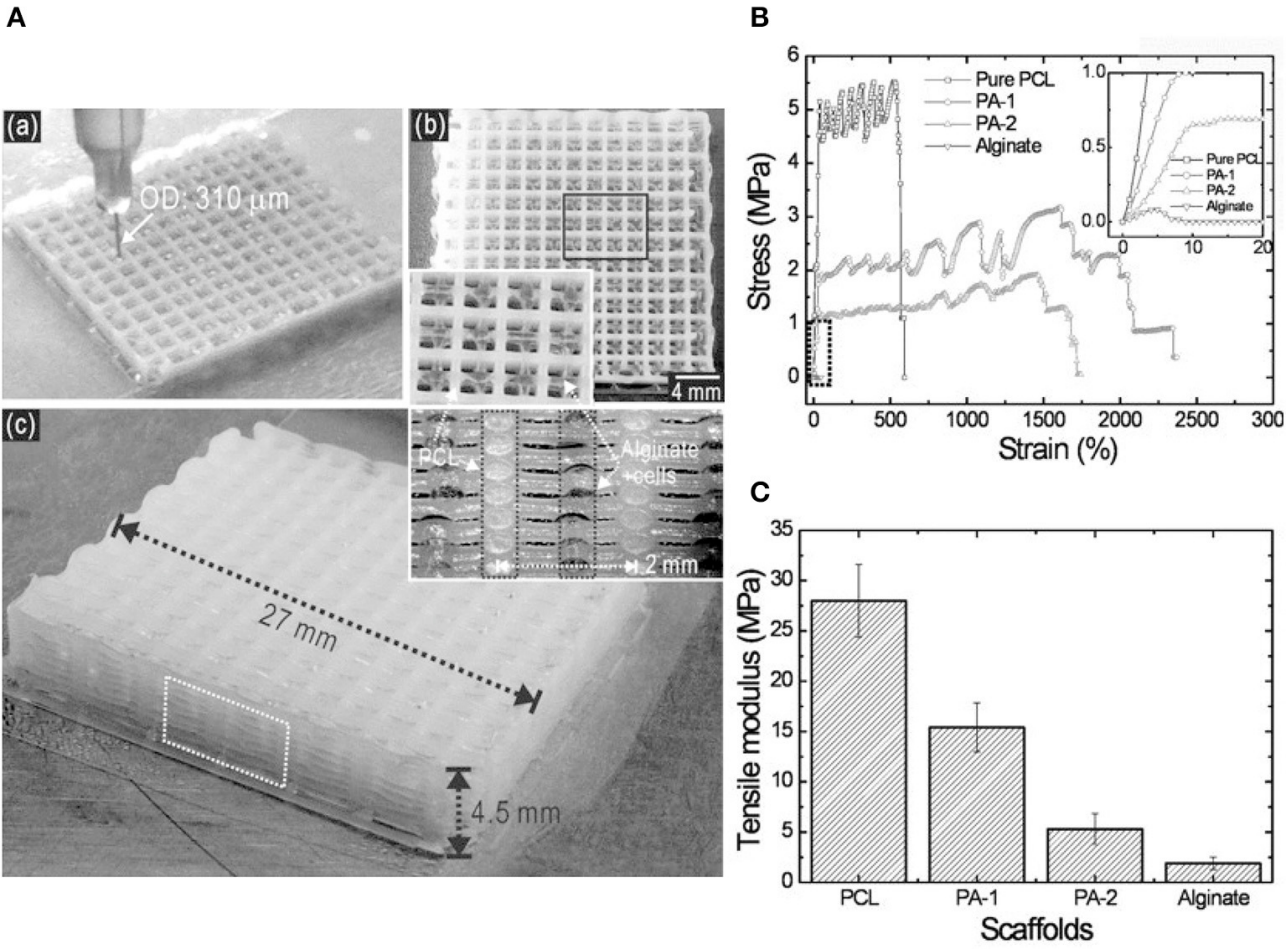
**(A)** Printing process (a), a surface image (b) and geometry shape (c) of hybrid scaffold consisting of cell-laden alginate struts and PCL struts in layers; **(B)** Stress-strain curves for pure PCL, two hybrid scaffolds (PA-1 and PA-2), and alginate alone. The curves for PCL and two hybrid scaffolds experienced a similar trend, demonstrating the function of mechanical reinforcement for PCL; **(C)** Comparison of tensile modulus for PCL, PA-1, PA-2, and alginate. Reprinted with permission from Lee et al. (2013). Copyright (2013) John Wiley and Sons.

Figure 10A presents a hybrid scaffold with a two-phase structure, consisting of both cell-laden alginate struts and PCL struts (Lee et al., 2013). As demonstrated by the stress-strain results of different formulations examined (Figure 10B), the PCL component could improve significantly the mechanical properties of the hybrid scaffolds, showing a similar characteristic to that of the pure PCL, which is significantly greater than that of the scaffold made of pure alginate. The substantial improvement (Figure 10C), e.g., tensile modulus from 2.5 MPa of alginate scaffolds alone to 15.4 MPa of PA-1 hybrid scaffolds, was attributed to the adhesion between PCL struts. It appeared that the large mesh space of the PCL layers was beneficial for transporting nutrients and signal factors between biologically active layers. In summary, the hybrid scaffold with thermoplastic reinforced structure met the requirements for hard tissue regeneration, with additional benefits contributed by the thermoplastic components.

**Figure 11:**
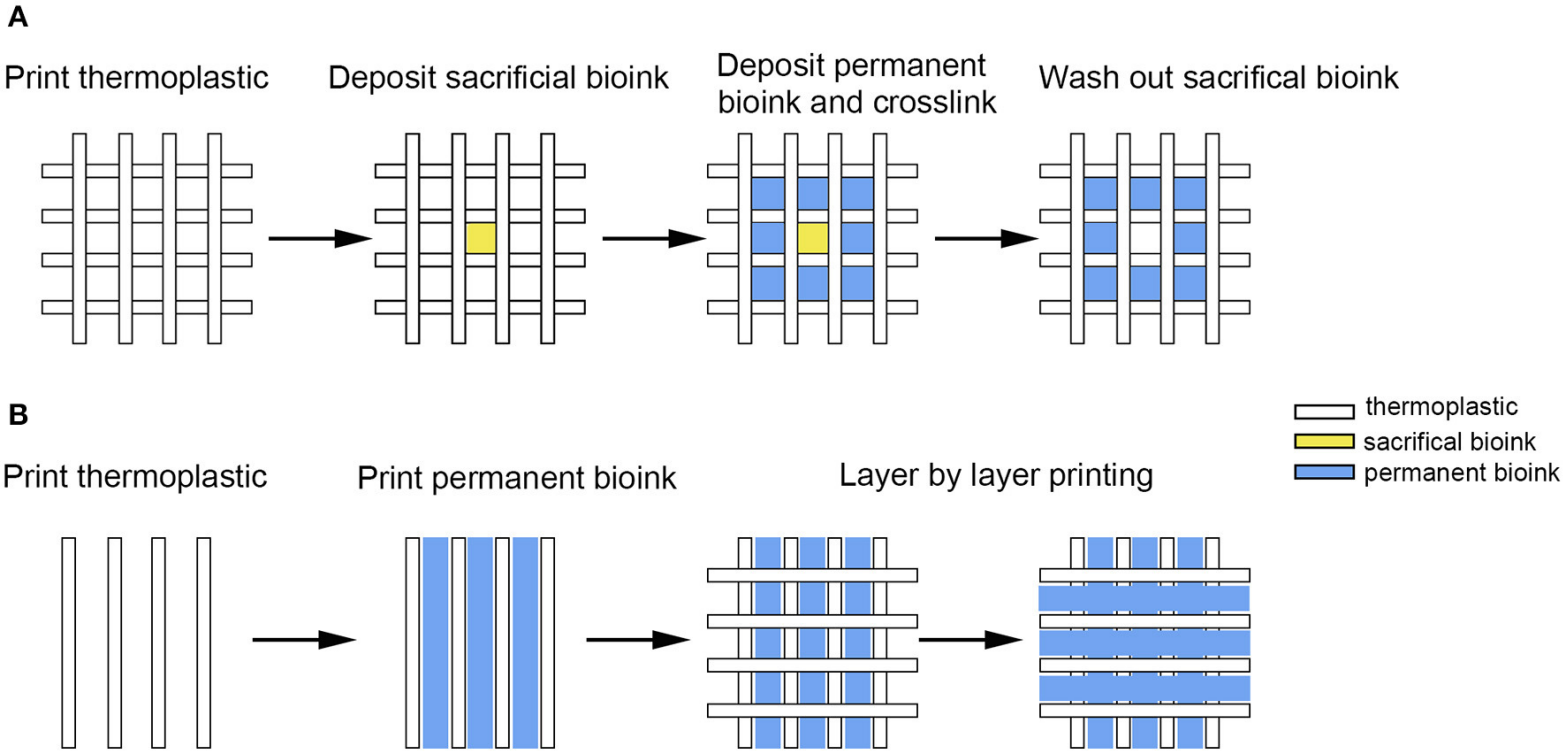
**(A)** Hydrogel-based bioink was deposited into the square area formed by 2 layers of thermoplastic; **(B)** Hydrogel with cells was deposited in the middle area of 2 thermoplastic filaments or customized shape to develop constructs with tailorable mechanical properties.

The other approach of integrating thermoplastic polymer with hydrogel is to construct a defined structure in a sequence, with individual components embedded, instead of the layered printing discussed. The embedding method offers an increased degree of flexibility: build a thermoplastic scaffold (Figure 11A), deposit hydrogel subsequently with the planned shape and structure (Figure 11B), and form a hybrid scaffold with tailorable mechanical properties (Schuurman et al., 2011).

It must be noted that some porous features should be planned in advance to facilitate the diffusion of small molecules required for various behaviors of the cells when a cell-containing hydrogel fills the thermoplastic structure (Daly and Kelly, 2019). Moreover, signal factors, DNA, and hydrogels with different functions can be placed in different regions of the same scaffold to mimic the complex microenvironment of a natural tissue.

To conclude, the aforementioned enhancement methods for bioink formulation have all been demonstrated with effectiveness but also limitations. Different natural tissues would have specific and customized requirements for the matching 3D scaffolding, which is challenging to achieve with one single enhancement strategy. It is therefore critical to design a bionic entity that couples several strategies in terms of printing technology, formulation, and post-processing to meet the requirements satisfactorily.

## Four-Dimensional Bioprinting

To implement one or even more “smart” features in a hydrogel scaffold, e.g., triggering mechanism upon external stimuli such as temperature, four-dimensional (4D) bioprinting method has been developed in recent years (Ashammakhi et al., 2018; Luo et al., 2019). The biomimetic scaffold contains components that are capable of responding toward either external environment or internal cells, which could either undergo immediate shape changes or may exhibit new functions over time to promote the behavioral expression of the encapsulated cells (Gao et al., 2016). The stimuli-responsive mechanism in the 4D bioprinting is not only a response function, but more importantly, a planned control of the geometrical, mechanical, and surface/interface characteristics to be achieved through reversible response, which makes the 4D bioprinting technology more suitable for the complex dynamics of natural tissues (Li et al., 2016; Sydney Gladman et al., 2016). The specific response could be physical transformations, surface magnetism, surface hydrophilicity/hydrophobicity, modulus, antibacterial properties, cell adhesion, growth factor inducing ability, and nutrient diffusion rate (Khoo et al., 2015; Ong et al., 2018).

**Figure 12:**
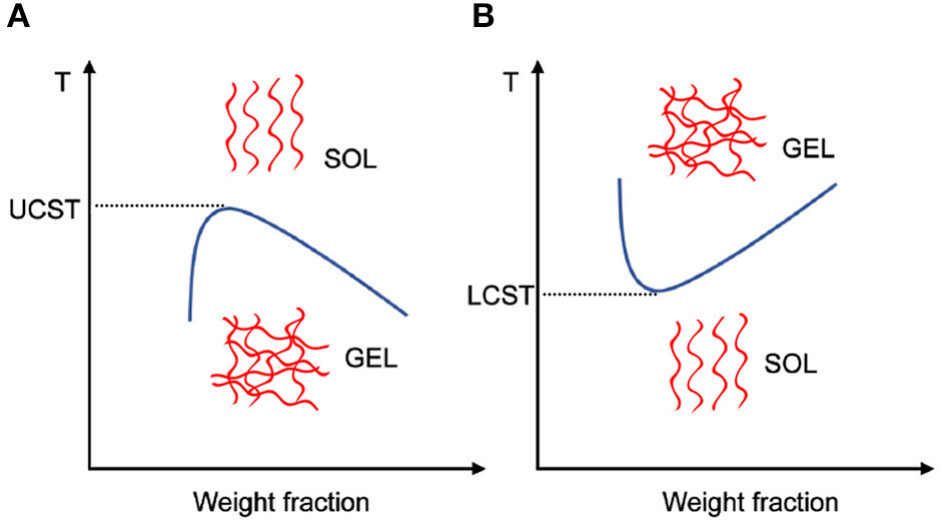
Schematic diagrams showing **(A)** UCST hydrogels and **(B)** LCST hydrogels experience gel–sol transition or sol–gel transition, respectively with an increased temperature. The blue lines represent the phase separation boundary.

According to the nature of the triggering mechanisms, the stimuli used in 4D bioprinting are categorized as physical stimuli such as temperature, electric field, magnetic field, acoustic, light, shear stress; chemical stimuli including pH, ions and gases; and biological stimuli such as enzymes (Koetting et al., 2015). Of the many available options, temperature is the most commonly used to induce behavior transformation for the bioprinted object. Schematic diagrams in Figure 12 demonstrate that the temperature-sensitive hydrogels such as poly(*N*-isopropylacrylamide), agarose and gelatin undergo a reversible volume change due to the collapse or expansion of the polymer chains in the solvent at lower critical solution temperature (LCST) or upper critical solution temperature (UCST) (Altomare et al., 2018).

Materials that are responsive to the pH of the surrounding medium have equally been widely used in tissue engineering – they are polyelectrolytes that contain functional groups such as carboxyl, sulfonic, phosphate, and pyridine (Dai et al., 2008; Wan et al., 2019). Based on the intra- and intermolecular forces that are controlled by the electrostatic interaction, polymer chains could change their configuration, e.g., from coil to globule when the charge of the functional groups is neutralized (Dai et al., 2008).

The same configurational transition mechanism can be applied for ion-responsive materials where the crosslinking and dissociation of the polymer chains are dependent on ions such as Ca^2+^ and Zn^2+^. Changing ion types or concentrations will affect the magnitude of the electrostatic interaction and consequently control the mechanical properties of the hydrogels (Zhou et al., 2013). As such, ion-responsive hydrogels can be used to adjust the rheology of bioinks before bioprinting and/or the mechanical properties of the constructs after bioprinting to generate a hierarchically organized bone structure (Krishnakumar et al., 2019), e.g., the responsiveness of alginate to Ca^2+^ has been widely applied in bioprinting (Rastogi and Kandasubramanian, 2019).

## Current Challenges and Future Prospects

Although reconstructing the structure and function of a defective bone tissue is an extremely complex process, bioprinting technique has shown a spectrum of great advantages in fabricating biomimetic implants, with significant progress being made in the selection of bioink components, the processibility of hydrogel-based bioink formulations, and the compatibility and mechanical properties of the biomimetic scaffold. However, bionic scaffolds are not as “intelligent” as natural tissues yet. It remains a pressing and significant challenge to improve the bioink formulation to improve the dynamic relationship between natural tissues, cells, and the environment. A comprehensive understanding of the composition of the specific osteoblast extracellular matrix (Lamers et al., 2010), the internal relationships and dynamic functions of the components will certainly provide underpinning principles for the design of future bioinks (Kashte et al., 2017).

It appears that an improved biocompatibility, using smart materials, will facilitate the differentiation and proliferation characteristics of the encapsulated stem cells (Anjum et al., 2016), and the vascularization of 3D scaffolds after implantation (Santos and Reis, 2010; Lee et al., 2014; He et al., 2019; Liu et al., 2020). The responsiveness of such smart materials can be reflected by a precise regulation of the release and binding of growth factors, which promotes the production of cell instructive matrices with tissue healing and regeneration functions, and contributes to the *in situ* vascularization of tissue repair. To improve the mechanical properties of the bionic bone tissue, one could consider a combinatorial approach of the enhancement strategies reviewed here: their synergistic effects warrant some in-depth investigations.

## Conclusion

Designing novel bioink formulations, in conjunction with using 4D bioprinting technology, to fabricate a scaffold with desired properties such as environmental sensitivity have shown significant progress and great potential. However, the complexity of the process, technique, and materials involved in reconstructing a defective bone tissue remain a challenging task. In this review, various requirements of the bioink/scaffold used for bone regeneration have been systematically discussed, and complemented with exemplar studies. The mismatched characteristics offered by the hydrogels used in the repair of hard tissues will certainly prompt the research communities to develop new solutions based on the existing reinforcement technologies. It is highly desirable to construct scaffolds replicating natural tissues in the ability of stem cell proliferation and differentiation, biomimetic material degradation, matrix remodeling, etc. Adding suitable smart materials to the bioink formulations could open up new opportunities to trigger shape transformation and adjust the aforementioned factors under specific environmental changes.

## Author Contributions

NL drafted the original manuscript with contributions by RG and ZZ. NL and ZZ contributed to the revision of the draft. ZZ and RG contributed to the supervision, validation, and funding acquisition. All authors contributed to the article and approved the submitted version.

## Conflict of Interest

The authors declare that the research was conducted in the absence of any commercial or financial relationships that could be construed as a potential conflict of interest.
